# Supplementary material for: Endovascular transplantation of mRNA-enhanced mesenchymal stromal cells results in superior therapeutic protein expression in swine heart
Source: Mol Ther Methods Clin Dev. 2024 Feb 27;32(2):101225. doi: 10.1016/j.omtm.2024.101225 (PMC10950887; doi:10.1016/j.omtm.2024.101225)
Supplement: Document S1. Figures S1–S3, Tables S1, and S2 [file mmc1.pdf]

OMTM, Volume *32*

Supplemental information

Endovascular transplantation of mRNA-enhanced mesenchymal stromal cells results in superior therapeutic protein expression in swine heart

Jonathan Al-Saadi, Mathias Waldén, Mikael Sandell, Jesper Sohlmér, Rikard Grankvist, Ida Friberger, Agneta Andersson, Mattias Carlsten, Kenneth Chien, Johan Lundberg, Nevin Witman, and Staffan Holmin

Supplemental figures

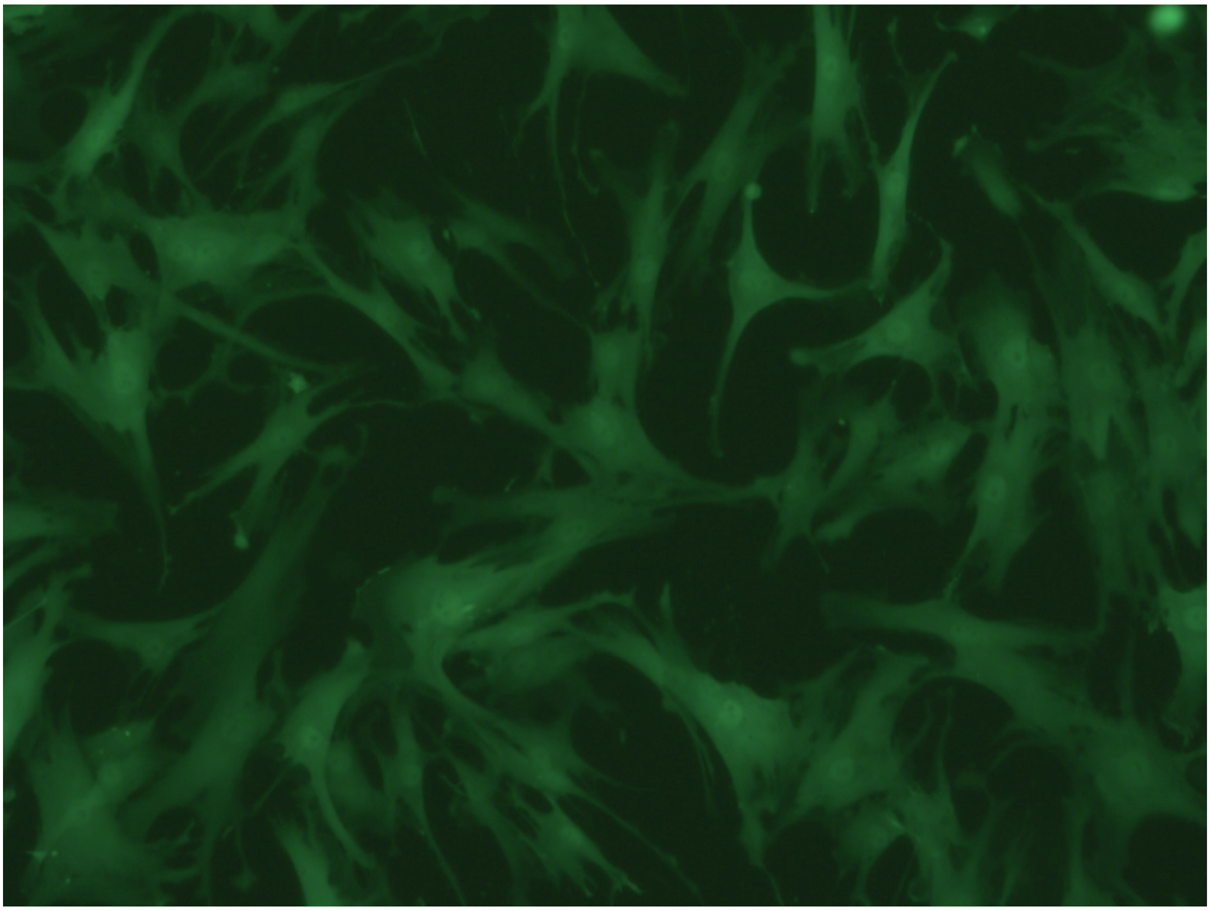

Figure S1. hMSCs electroporated with 20 μg modGFP per million cells at 7 days after electroporation.

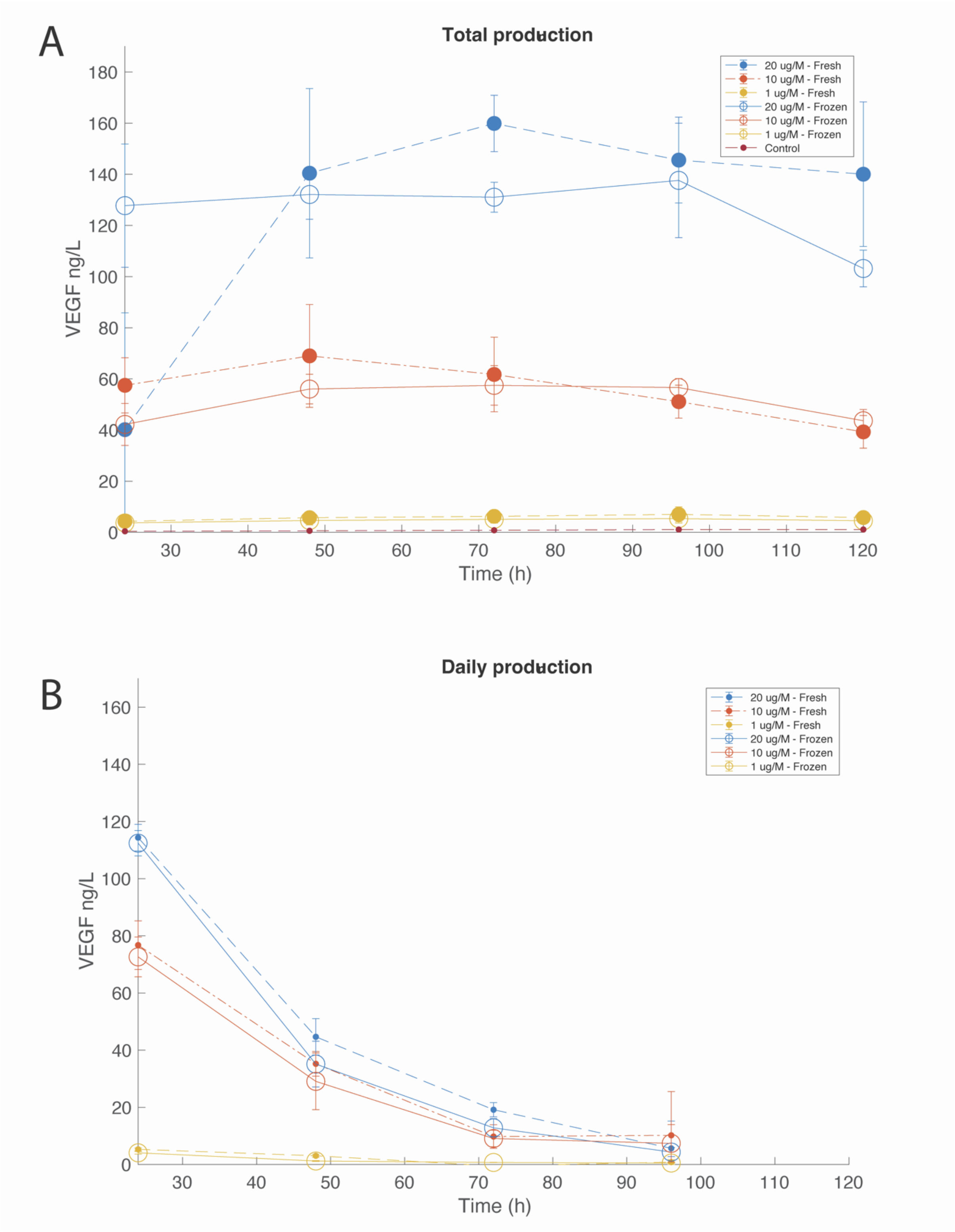

Figure S2. Levels of VEGF-A protein expression in cell culture supernatants of pre- and post-freeze-thawed hMSCs. A. Total production. B. Daily production. Triplicates in each group, $p > 0.05$.

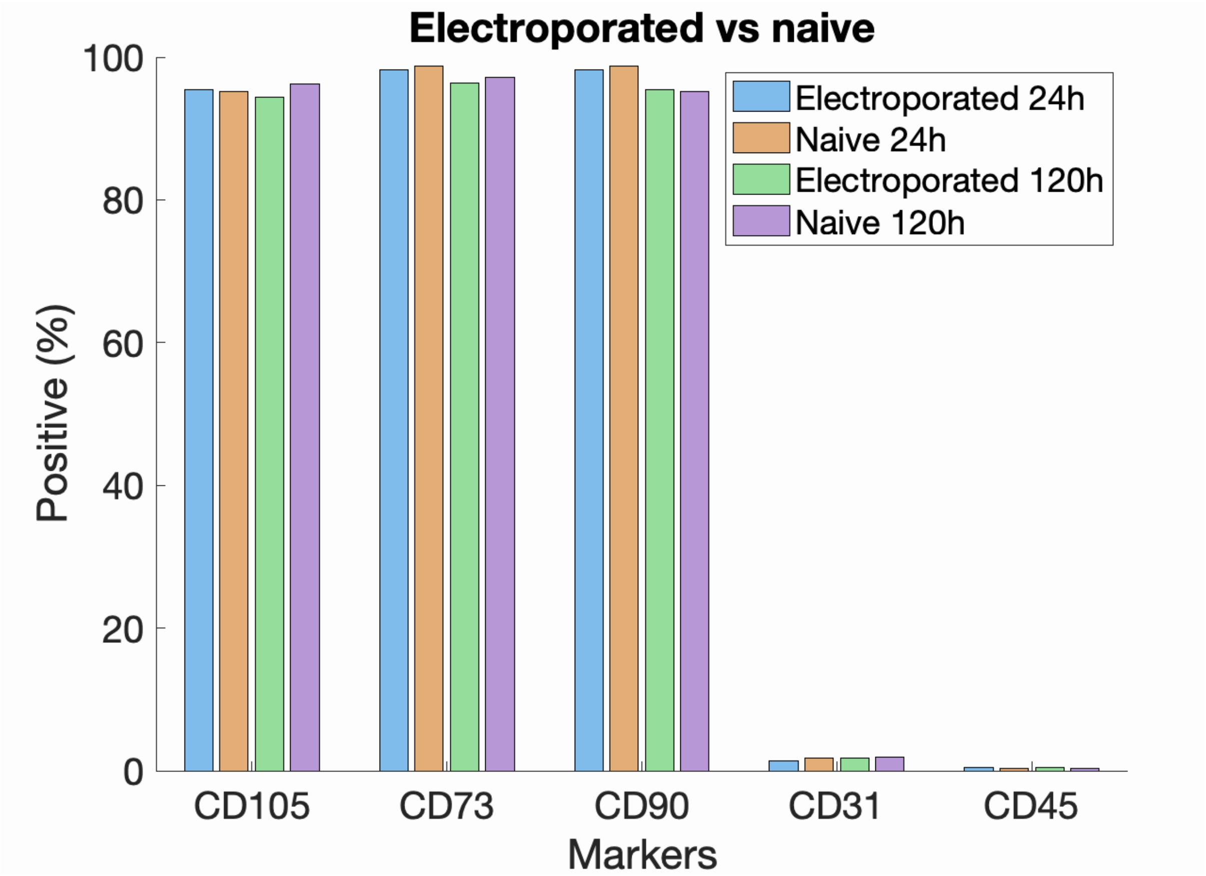

Figure S3. Quantification of hMSC markers pre- and post- electroporation monitored at 24h and 120h using flow cytometry. Triplicates in each group, $p > 0.05$.

Table S1. Flushed cells through the catheter at different speeds and concentrations.

Cell concentration (cells/uL)	Time after thaw (h)	Viability, mean (SD)	Flow speed (uL/s)	Proportion flushed, mean (SD)
30 000	6	88% (6%)	1.67	89% (8%)
50 000	0	86% (6%)	6.67	91% (7%)
	0	92 (6%)	1.67	80% (18%)
	0	94% (5%)	0.83	62% (15%)
20 000	0	89% (2%)	6.67	86% (7%)
	0	95% (5%)	1.67	61% (8%)
	0	95% (3%)	0.83	55% (9%)

Table S2. Open reading frame sequences used for modRNA production.

Name	Sequence
GFP	atggtgagcaagggcgaggagctgttcaccggggtggtgcccatcctggtcgagctggacgg cgacgtaaacggccacaagttcagcgtgtccggcgagggcgagggcgatgccacctacggca agctgaccctgaagttcatctgcaccaccggcaagctgcccgtgccctggcccaccctcgtg accaccctgacctacggcgtgcagtgcttcagccgctaccccgaccacatgaagcagcacga cttcttcaagtccgccatgcccgaaggctacgtccaggagcgcaccatcttcttcaaggacg acggcaactacaagacccgcgccgaggtgaagttcgagggcgacaccctggtgaaccgcatc gagctgaagggcatcgacttcaaggaggacggcaacatcctggggcacaagctggagtacaa ctacaacagccacaacgtctatatcatggccgacaagcagaagaacggcatcaaggtgaact

	tcaagatccgccacaacatcgaggacggcagcgtgcagctcgccgaccactaccagcagaac<br>acccccatcggcgacggccccgtgctgctgcccgacaaccactacctgagcacccagtccgc<br>cctgagcaaagaccccaacgagaagcgcgatcacatggtcctgctggagttcgtgaccgccg<br>ccgggatcactctcggcatggacgagctgtacaagtaa
Human VEGF-A_{165}	atgaactttctgctgtcttgggtgcattggagccttgccttgctgctctacctccaccatgc<br>caagtggtcccaggctgcacccatggcagaaggaggagggcagaatcatcacgaagtggtga<br>agttcatggatgtctatcagcgcagctactgccatccaatcgagaccctggtggacatcttc<br>caggagtaccctgatgagatcgagtacatcttcaagccatcctgtgtgcccctgatgcgatg<br>cgggggctgctgcaatgacgagggcctggagtgtgtgcccactgaggagtccaacatcacca<br>tgcagattatgcggatcaaacctcaccaaggccagcacataggagagatgagcttcctacag<br>cacaacaaatgtgaatgcagaccaaagaaagatagagcaagacaagaaaatccctgtgggcc<br>ttgctcagagcggagaaagcatttgtttgtacaagatccgcagacgtgtaaatgttcctgca<br>aaaacacagactcgcgttgcaaggcgaggcagcttgagttaaacgaacgtacttgcagatgt<br>gacaagccgaggcggtga
