# Supplementary material for: Endovascular transplantation of mRNA-enhanced mesenchymal stromal cells results in superior therapeutic protein expression in swine heart
Source: Mol Ther Methods Clin Dev. 2024 Feb 27;32(2):101225. doi: 10.1016/j.omtm.2024.101225 (PMC10950887; doi:10.1016/j.omtm.2024.101225)
Supplement: Document S2. Article plus supplemental information [file mmc2.pdf]

Molecular Therapy
Methods & Clinical Development
Original Article

Endovascular transplantation of mRNA-enhanced mesenchymal stromal cells results in superior therapeutic protein expression in swine heart

Jonathan Al-Saadi,[1,2,3] Mathias Waldén,[1] Mikael Sandell,[1,3,4] Jesper Sohlmér,[5] Rikard Grankvist,[1] Ida Friberger,[1] Agneta Andersson,[6] Mattias Carlsten,[6,7] Kenneth Chien,[5] Johan Lundberg,[1,2,3] Nevin Witman,[1] and Staffan Holmin[1,2,3]

[1]Department of Clinical Neuroscience, Karolinska Institute, Tomtebodavägen 18A, 171 65 Stockholm, Sweden; [2]Department of Neuroradiology, Karolinska University Hospital, 171 64 Stockholm, Sweden; [3]MedTechLabs, Stockholm, Sweden; [4]Division of Micro and Nanosystems, KTH Royal Institute of Technology, Malvinas väg 10, 114 28 Stockholm, Sweden; [5]Department of Cell and Molecular Biology, Karolinska Institute, Solnavägen 9, 171 65 Stockholm, Sweden; [6]Department of Medicine, Huddinge, Center for Hematology and Regenerative Medicine, Karolinska Institutet, Stockholm, Sweden; [7]Center for Cell Therapy and Allogeneic Stem Cell Transplantation, Karolinska Comprehensive Cancer Center, Karolinska University Hospital, Stockholm, Sweden

Heart failure has a poor prognosis and no curative treatment exists. Clinical trials are investigating gene- and cell-based therapies to improve cardiac function. The safe and efficient delivery of these therapies to solid organs is challenging. Herein, we demonstrate the feasibility of using an endovascular intramyocardial delivery approach to safely administer mRNA drug products and perform cell transplantation procedures in swine. Using a *trans*-vessel wall (TW) device, we delivered chemically modified mRNAs (modRNA) and mRNA-enhanced mesenchymal stromal cells expressing vascular endothelial growth factor A (VEGF-A) directly to the heart. We monitored and mapped the cellular distribution, protein expression, and safety tolerability of such an approach. The delivery of modRNA-enhanced cells via the TW device with different flow rates and cell concentrations marginally affect cell viability and protein expression *in situ*. Implanted cells were found within the myocardium for at least 3 days following administration, without the use of immunomodulation and minimal impact on tissue integrity. Finally, we could increase the protein expression of VEGF-A over 500-fold in the heart using a cell-mediated modRNA delivery system compared with modRNA delivered in saline solution. Ultimately, this method paves the way for future research to pioneer new treatments for cardiac disease.

INTRODUCTION

Heart failure is an advanced stage of cardiovascular disease.[1] It remains one of the foremost causes of death in the world.[2] Preclinical studies and clinical trials have investigated regenerative therapies, such as gene therapy or cell transplantation, to improve cardiac function.[3–6] Although decades of extensive, predominantly preclinical studies have yielded attractive results, neither approach has yet become a clinical standard of care. Novel delivery systems are needed to further improve and enhance genetic therapies and stem cell transplantation to solid organs.

To rehabilitate cardiac function, several cell-based therapies are under investigation. A multitude of different cell types have been employed, including human mesenchymal stem cells (hMSCs).[3,7–9] Additionally, studies have shown that stem cell-derived cardiomyocytes can remuscularize the heart and enhance cardiac function in large animal models.[10,11] These studies and others have shown that repopulating damaged myocardium with exogenous cells come at the cost of inducing ventricular arrhythmias, which is the foremost cause of death in heart failure patients.[2] The expectation that cells generate or differentiate into functional and synchronized cardiomyocytes in severe inflammatory or hypoxic conditions is optimistic. Recent findings suggest that the primary benefits of cell-based therapies arise from their paracrine effects, including immunomodulation, angiogenesis, and anti-apoptotic signals.[12,13]

hMSCs have emerged as a potential cell therapy for heart failure due to their beneficial immunomodulatory effects.[14] Several previous and ongoing clinical trials have examined the effects of hMSCs for cardiac indications, the results of which are often unexceptional.[15,16] The limited therapeutic efficacy observed in these systemic approaches can be attributed to first-pass effects and lung entrapment post-infusion.[17] To overcome these challenges, targeted local administration could be advantageous. A systemic approach might lead to dilution of MSC-produced cytokines and potential off-target effects.

Received 21 June 2023; accepted 23 February 2024;
https://doi.org/10.1016/j.omtm.2024.101225.

Correspondence: Johan Lundberg, Department of Clinical Neuroscience, Karolinska Institute, Tomtebodavägen 18A, 171 65 Stockholm, Sweden.
E-mail: j.lundberg@ki.se
Correspondence: Nevin Witman, Department of Clinical Neuroscience, Karolinska Institute, Tomtebodavägen 18A, 171 65 Stockholm, Sweden.
E-mail: nevin.witman@ki.se
Correspondence: Staffan Holmin, Department of Clinical Neuroscience, BioClinicum J5:20, Akademiska stråket 1, Karolinska Universitetssjukhuset, 171 64 Solna, Sweden.
E-mail: staffan.holmin@ki.se

This is an open access article under the CC BY license (http://creativecommons.org/licenses/by/4.0/).

Conversely, a targeted approach could ensure prolonged persistence and a higher concentration of paracrine factors at the target site. Specifically, for cardiac therapy, the endocardium acts as a barrier between the myocardium and blood, raising questions about the effectiveness of systemically administered cells or cytokines in reaching the heart muscle.

Gene therapy is another potential treatment for heart failure. Unlike cell transplantation, gene therapy approaches face many challenges with regard to delivering genetic material to cells *in vivo*.[18] Initially, studies focused on injecting naked DNA plasmids, but have shifted to other vectors like lipofectamine or viral systems.[19,20] The use of non-integrating gene therapy in which the DNA remains separate from the genome and is not expressed permanently was speculated to be less harmful and not cause serious inflammatory responses.[21] Integrating vectors on the other hand insert viral DNA into the host genome increasing the risk for insertional mutagenesis, and overt immune responses due to preformed antibodies.[22] This raises safety and efficacy concerns.[23]

Chemically modified mRNA (modRNA) has emerged as a novel approach to express proteins *in vitro* and *in vivo*.[24] The impact of incorporating nucleoside base modifications to mitigate innate immune responses from exogenously delivered mRNA has undoubtedly helped to unlock the potential of mRNA therapeutics.[24] The novelty, safety, and efficacy of an mRNA-based drug platform has gained wide attention in light of the Pfizer/BioNTech and Moderna SARS-CoV-2 mRNA vaccines.[25,26] Unlike DNA plasmids or viral vectors, delivery of modRNA results in a transient and high burst of protein expression when delivered *in vivo,* which may be ideal for driving the expression of paracrine signals.[27] In addition, modRNA delivery bypasses many inflammatory pathways in the cells, such as the toll-like receptors.[24,28] One major caveat in working with modRNA is expense related, as the use often requires the application of large doses to generate strong physiological effects.[29] More recently, Ai et al. and Yu et al. have applied a cell-mediated modRNA delivery system using enriched cells with modRNA encoding for vascular endothelial growth factor (modVEGF) to treat heart failure and peripheral vascular disease, respectively, which required considerably lower doses of modRNA.[30,31]

The delivery of cell therapy products or genetic materials has previously required the use of large needles in thoracotomy or intra-coronary infusion, yielding mixed results.[32] Thoracotomy, an invasive procedure, is in decline.[33] Intravascular infusion limits usable cell types and often results in systemic delivery, dispersing cell products to spleen, liver, and lung.[17,34,35] We have designed an endovascular catheter for *trans*-vessel wall delivery granting direct organ access.[36] The *trans*-vessel wall device (TW device) has a small internal diameter, posing cell delivery challenges due to shear stress during injection but limiting reflux due to a smaller injection hole.

In this proof-of-concept study, we present a cell-mediated delivery method of modRNA, using an endovascular technique, with the possibility to transiently deliver, with significant expression, any protein of choice locally in the heart.

RESULTS

Naive hMSCs can be electroporated with modRNA to express VEGF-A and GFP

To deliver modRNA-enhanced hMSCs to the swine heart using the TW device, we first sought to optimize the modRNA concentrations for cell loading. We used large-scale electroporation techniques and conducted experiments with naive hMSCs, dosing them with modGFP and modVEGF at three different concentrations. We found no significant difference in viability after electroporation with 1 μg, 10 μg, or 20 μg modGFP per 1 million cells (86.7% ± 2.52%, 86.44% ± 7.33%, 83.44% ± 3.56%, $p = 0.67$). However, there was a significant increase in the proportion of cells that expressed GFP between the groups that was respective to dose (77.27% ± 1.05%, 83.92% ± 6.2%, 90.78% ± 3.52%, $p < 0.001$, Figure 1A). Higher doses of modGFP also significantly increased mean fluorescent intensity (11.4 ± 1.1, 585.7 ± 40.6, 5,996% ± 2,173%, 12,790 ± 2,580, $p < 0.001$, $r^2 = 0.92$, Figure 1B). We also monitored the GFP signal by fluorescent microscopy (Figure 1C). The modGFP electroporated hMSCs remained fluorescent for at least 7 days after electroporation (Figure S1). In addition, we found that increasing doses of modVEGF resulted in increased daily and total VEGF-A secretion by plated hMSCs at all time points evaluated in both freeze-thawn and fresh cells ($p < 0.001$ and $p = 0.007$, respectively, Figures 1D and 1E; Figure S2). Of importance, hMSCs maintained their expression of hMSC markers at both 1 day and 5 days post electroporation (Figure S3).

modRNA-enhanced hMSCs have high viability and continue to express proteins after being flushed through the catheter

Next, to assess the optimal conditions for flushing the cells through the catheter, we designed a series of *in vitro* catheter studies where we thawed and flushed a total volume of 0.2 mL naive or modRNA-enhanced hMSCs through the TW device followed by immediate plating (Figure 2A). First, we flushed naive hMSCs at three different cell concentrations (50,000, 30,000, and 20,000 cells/μL) and three different flow speeds (30, 120, and 240 s). The proportion of flushed cells, i.e., the percent of cells that could pass through the TW device, was greater at high flow speeds and concentrations. At all concentrations, lower flow speeds tended to reduce the proportion of flushed cells, but the difference was not statistically significant (Figure 2B). The mean viability was consistently over 85% in all concentrations and flow speeds measured (Table S1). However, slower flow speeds increased the viability to above 90% (Figure 2C). Finally, to simulate extreme conditions, naive hMSCs were stored at room temperature for 6 h at a concentration of 30,000 cells/μL and then flushed through the catheter. The proportion of flushed cells was 89% ± 8%, with viability at 88% ± 6%. Next, we tested whether flushing cells through the catheter would have any negative effects on protein expression and secretion stemming from modRNA-enhanced hMSCs. Interestingly, catheter flushed modVEGF-enhanced hMSCs expressed VEGF to a similar extent to non-flushed modVEGF-enhanced cells at all time points (Figure 2D). Plated modGFP-enhanced cells at 24 h post catheter flush visually appeared similar to non–catheter-flushed cells under a fluorescent microscope (Figure 2E).

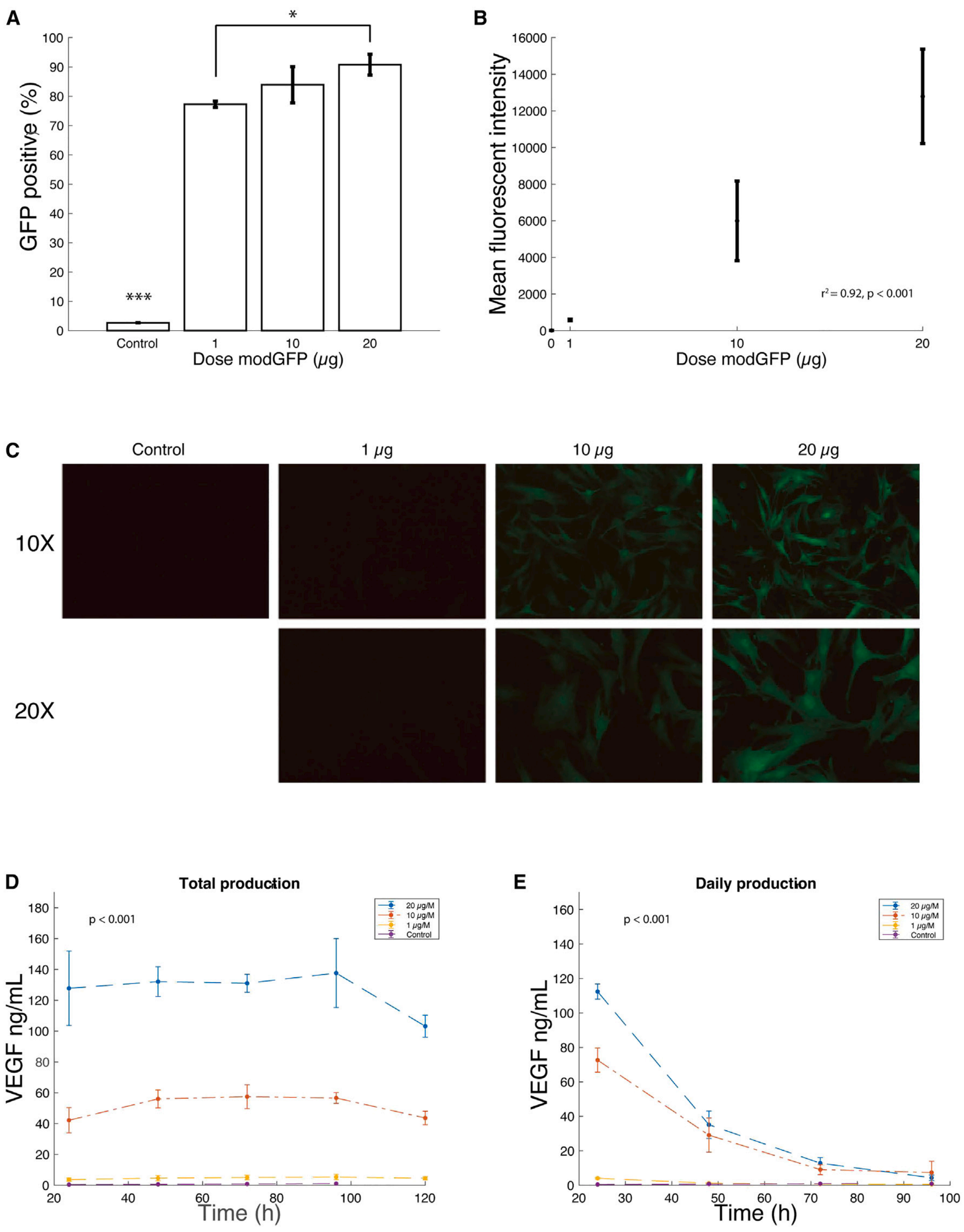

Figure 1. Naive hMSCs can be electroporated with modRNA to express GFP and VEGF protein

(A and B) The proportion of live GFP-positive cells and fluorescent intensity as assessed by flow cytometry after 24 h. One asterix (*) indicates $p < 0.05$ and three asterisks (***) indicate $p < 0.001$. (C) Fluorescent images of cells 24 h after electroporation show higher GFP expression with higher dose modGFP. (D and E) Daily and total production of VEGF-A *in vitro* was higher with higher dose modVEGF. Error bars represent the standard deviation.

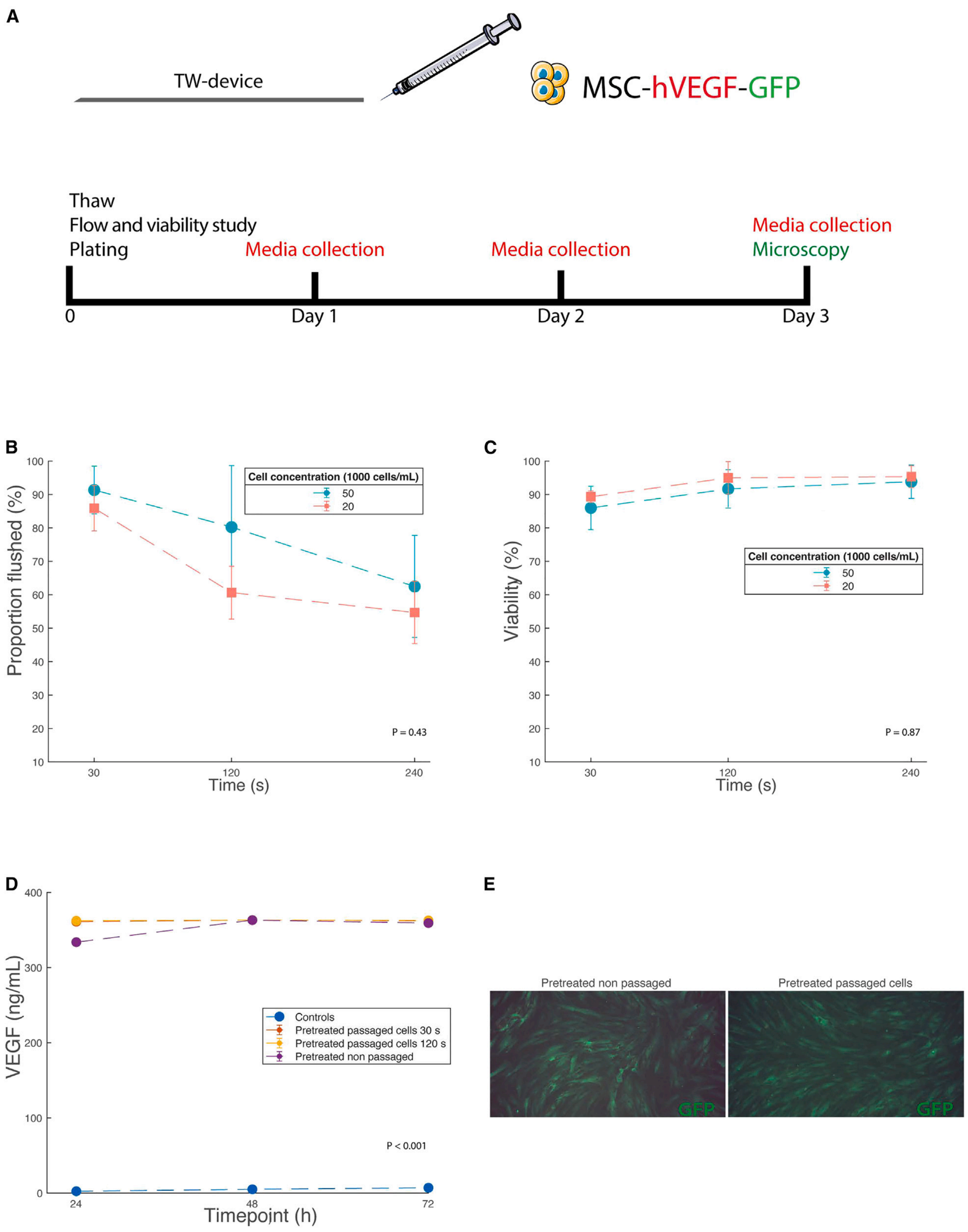

(legend on next page)

Endovascularly transplanted hMSCs to the myocardium using the TW device are found 24 h and 72 h post-injection in the swine heart

To explore the feasibility of hMSC transplantation to the heart with the TW device, we injected six swine with 6 million naive hMSCs in six sites, for a total of 36 million cells around the apex of the heart, in the absence of immunosuppressants (Figures 3A and 3B). Animals were euthanized at 24 h (n = 3) and 72 h (n = 3) post-injection (Figure 4A).

All injection sites were identified through macroscopic analysis of the tissue. The median number of cells per slide found around the injection site was 127.5 (IQR ±50.5) 1 day post-injection and 81 (IQR 106) 3 days post-injection (Figure 4B). Immunohistochemical analysis revealed hMSCs were mostly localized to areas immediately around the injection sites (Figure 4C).

Macroscopic analysis showed signs of microhemorrhages and edema 24 h post-injection. At 72 h post-injection, granulation tissue had been formed at the injection sites (Figure 4D). We found no other significant structural damage. The pericardial sack was intact and the pericardial fluid was transparent, i.e., no tamponade. Histological analysis revealed a disruption of the tissue structure and integrity at the injection sites with focal loss of cardiomyocytes. There was cytoplasmic eosinophilia of the cardiomyocytes and microscopic bleeding adjacent to the injection sites. Some cardiomyocytes at the injection sites showed signs of myofiber vacuolation at 24 h post injections and loss of cellular and nuclear details 72 h post injections. Granulocyte and monocyte infiltration was observed around the injection sites (Figure 4D).

modRNA-enhanced cells increase the protein expression of VEGF in the heart

Intrigued by the possibility of transplanting the hMSCs with the TW device with no immunomodulation, we next sought to examine the possibility of using a cell-mediated approach to deliver and express modRNA in the heart. Therefore, we designed an experiment where swine either received naive hMSCs, modVEGF-enhanced hMSCs, or modVEGF mRNA in a citrate-saline buffer. The animals were euthanized 24 h (n = 9) and 72 h (n = 3) post injections, and levels of VEGF-A were quantified at the injection sites (Figure 5). The *in vivo* production of VEGF-A protein following the administration of modVEGF-enhanced hMSCs was significantly higher compared with other groups at all timepoints. One day post-injection, the modVEGF-enhanced hMSCs exhibited VEGF-A levels of 41.1 ± 15.7 μg/mL/g, surpassing the naive MSCs (0.147 ± 0.03 μg/mL/g) and modVEGF-only groups (0.127 ± 0.09 μg/mL/g) by over 200 times. This difference was statistically significant ($p < 0.001$). Notably, both the naive hMSCs and the saline-formulated modVEGF groups showed detectable, albeit low, levels of human VEGF-A protein *in vivo*, but these levels did not differ significantly from each other. Three days after the injections, there was a general decrease in VEGF-A protein levels across all groups. However, the modVEGF-enhanced hMSCs still demonstrated a markedly higher VEGF-A production (0.79 ± 0.31 μg/mL/g), approximately 500 times greater than that of the naive MSCs (0.0017 ± 0.0002 μg/mL/g) and the modVEGF-only groups (0.0016 ± 0.0002 μg/mL/g), a difference that was again statistically significant ($p < 0.001$).

DISCUSSION

This proof-of-concept study explored the possibility of delivering modRNA through a cell-mediated approach directly to the myocardium using endovascular techniques. Herein, we demonstrate that subjecting the hMSCs to different flow rates and concentrations upon flushing with the TW device marginally affects cell viability and protein expression of modRNA-enhanced cells. It is likely that the shear forces applied to the hMSCs being passed at alternative speeds, volumes, and concentrations play a critical role in determining the overall viability of the cells, and may need to be carefully optimized for cell-specific subtypes.[9] The transplantation of hMSCs using the TW device resulted in localized cellular penetrance to areas near the injection sites within the myocardium for at least 72 h following administration. This is a surprising feat considering the animals received no immunomodulation. Furthermore, microscopic evaluation of the injected tissue pieces revealed minimal impact on tissue integrity with this approach. The TW device is designed to give minimal trauma to the tissue and allow for multiple rapid injections to cover a large part the myocardium with easy navigation. Finally, we could increase the protein expression of VEGF-A over 200-fold in the heart at 1 day post-injection and almost 500-fold after 3 days using a cell-mediated modRNA delivery approach compared with modRNA delivery in a citrate-saline buffer. These results indicate a promising method for targeted regenerative therapies in the heart for treating heart failure or infarction in patients. The intervention shown here could easily be adopted into routine clinical endovascular techniques and most likely increase the local expression of any protein of choice in heart tissue and alternative solid organs.

Previous studies have focused on injecting naked modRNA in a saline formulation or complexed mRNA with lipofection-based methods.[27,29,37] To establish therapeutic efficacy, saline-formulated modRNA requires large doses. For example, extrapolating the doses from rodent studies to humans would require approximately 167 mg of modRNA to achieve physiological effects.[29] Ongoing clinical trials are required to use large doses, 3 mg per injection, and up to 30 injections in the heart during open thoracotomy.[38] This is costly and highly invasive. Compared with similar studies, our method increased the VEGF-A concentration substantially using the TW device alone when injecting naked modVEGF in a saline citrate solution and even further using the cell-mediated approach combined with the TW device.[29,37,39]

Figure 2. modRNA-enhanced hMSCs have a high viability and continue to express proteins after catheter pass-through

(A) Schematic image of experimental set up. (B) Comparison of flow speeds and the proportion of cells at different concentrations that were flushed through the TW device. (C) The relation of flow speeds to viability. (D) VEGF levels of flushed modRNA-enhanced cells. (E) Fluorescent microscopy images of modRNA-enhanced hMSCs after flush with the TW device compared with non-flushed cells. Error bars represent the standard deviation.

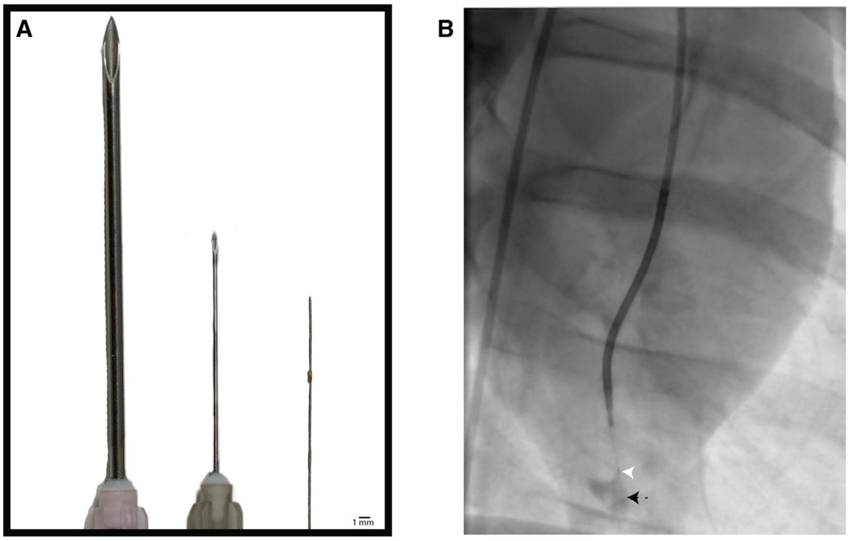

Figure 3. The TW device is smaller compared with other needles and can inject substances into the myocardium endovascularly

(A) The TW device (far right) compared with a 26-gauge (G) needle (middle) and a 18G needle (far left). (B) Fluoroscopic image of the TW device (white arrow) being used to inject contrast (black arrow) into the left ventricular apex.

Several targeted catheter-based delivery systems exist.[36] Notably, Järveläinen et al. employed an alternative catheter system, the NOGA-myostar to deliver modVEGF in a saline solution directly to ischemic myocardium.[40] This did not lead to meaningful transduction of VEGF in the myocardium, and leads us to speculate that the effects demonstrated herein can not be solely explained by using a device for direct myocardial delivery. We speculate that the TW device, since it is smaller than the NOGA system, allows for higher local concentration due to increased tissue retention by avoiding back-leakage through the injection channel. Furthermore, the cell-mediated approach removes the need to depend on cardiomyocytes to express the protein and might allow for further protection of the VEGF modRNA upon delivery. As previously suggested, the cell-mediated delivery approach of modRNA leads to enhanced stability and protein production.[30] The increased protein expression could translate to greater physiological effects or allow for lower doses of modRNA, which could reduce manufacturing costs.

In this study, we used electroporation to transfect the cells with modRNA. Growing evidence suggests that transfection agents such as lipofectamine can trigger immunological responses in the cell or host as well as change the fate of hMSCs.[41,42] Therefore, a major benefit of electroporation is that no transfection agent is needed. When comparing the protein expression, the levels seem similar or higher to other methods.[27,29,37] In line with similar studies, we could find the xenologous transplanted hMSCs with no immunomodulation at least 72 h after injection.[43]

Naive hMSCs secrete VEGF-A natively.[44] Interestingly, we found that the levels of human VEGF-A in the swine cardiac tissue were not significantly different in the animals receiving naive hMSCs compared with naked modVEGF. We speculate that a dose greater than the 60 μg of modVEGF per injection site chosen for this study, would have yielded a significant difference. However, we believe this result might demonstrate the power of harnessing immune-silent cells to deliver therapeutically sufficient levels of *in vivo* produced protein via modRNA. Such a system could overcome the safety concerns with regard to high-dose administrations or the previous challenges facing repeat dosing.

One of the limitations of this study is that we performed the experiments in healthy swine. Future research should evaluate this approach in heart failure models. We speculate that the difference in protein expression would be even higher because of the diseased myocardium's reduced ability to express proteins. Finally, it would be interesting to examine if the system reported herein, namely the delivery of mRNA via a cell-mediated approach using a TW device, could in fact improve relevant clinical endpoints in heart failure models.

In summary, many exploratory preclinical studies within the cell and gene therapy space are uncovering novel and exciting results for heart failure treatment, but have yet to be incorporated into clinical practice. It is evident that these regenerative methods could be beneficial in treating heart failure. However, the delivery of cells and genes is a significant outstanding issue to solve. Here, we explore the possibilities of using cell-mediated modRNAs for local solid organ protein delivery. In conclusion, the TW device can transplant modRNA-enhanced hMSCs to the myocardium using minimally invasive endovascular techniques. This cell-mediated approach yields grossly higher VEGF-A concentration in the tissue than modRNA delivered in a physiologically relevant saline solution. This study has highlighted the possibility of overcoming critical issues in cell and gene delivery and the applicability of using protein expression manipulation as a promising future treatment of cardiac failure.

METHODS

Chemically modified mRNA synthesis and formulation

Synthesis of modRNA was performed by *in vitro* transcription of a linearized DNA template using T7 RNA polymerase. The template incorporated generic 5′ and 3′UTRs and a poly-A tail, as previously described.[31] During the *in vitro* transcription reaction, uridine was replaced with N1-methylpseudouridine. RNA was purified using Ambion MEGAclear (Ambion, Austin, TX, USA) spin columns and treated with Antarctic phosphatase (New England Biolabs, Ipswich, MA, USA) for 30 min at 37 °C to remove residual 5′-phosphates. The RNA was repurified and quantified by Nanodrop (Thermo Scientific, Waltham, MA, USA) and then resuspended in 10 mM Tris HCl,

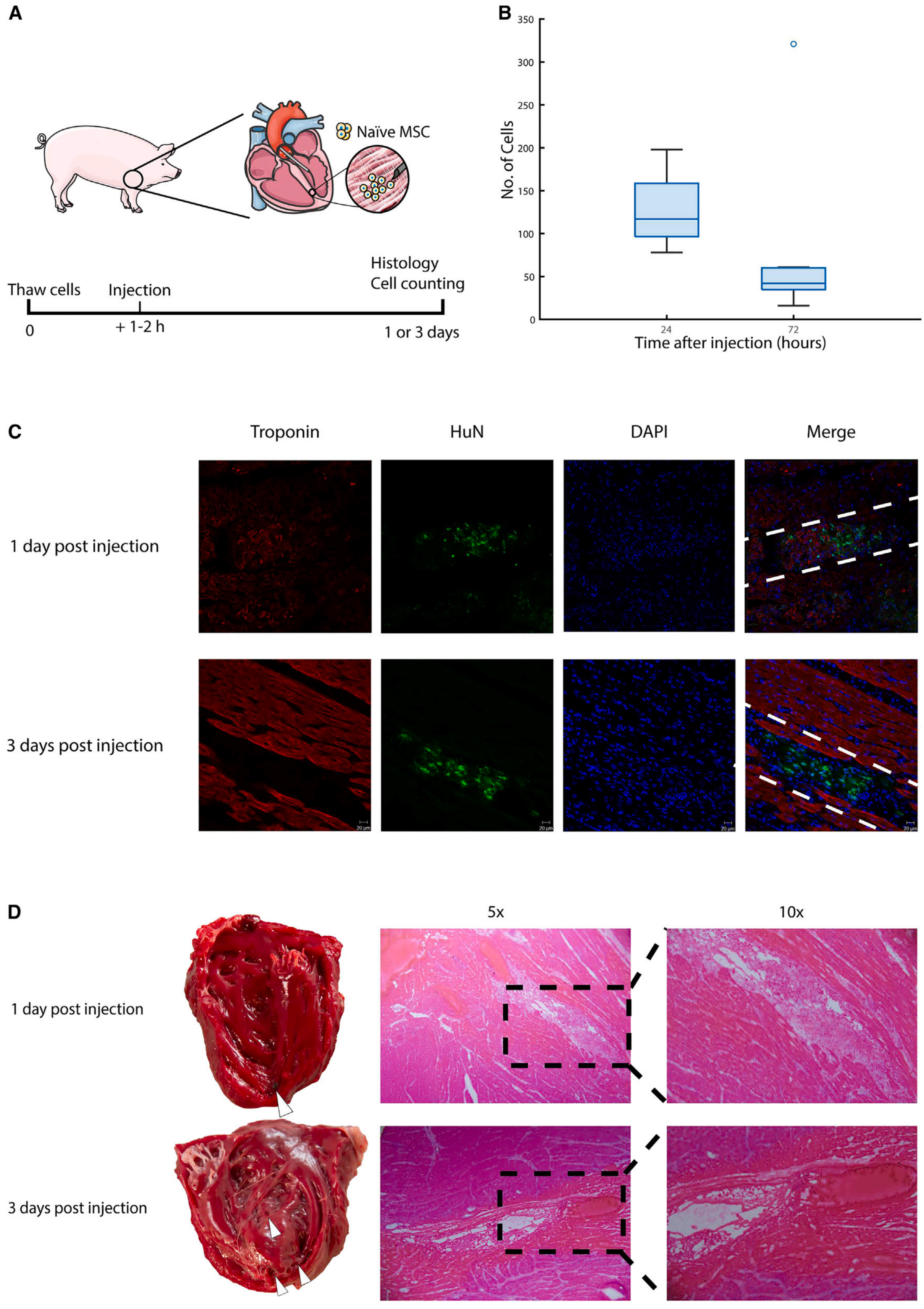

(legend on next page)

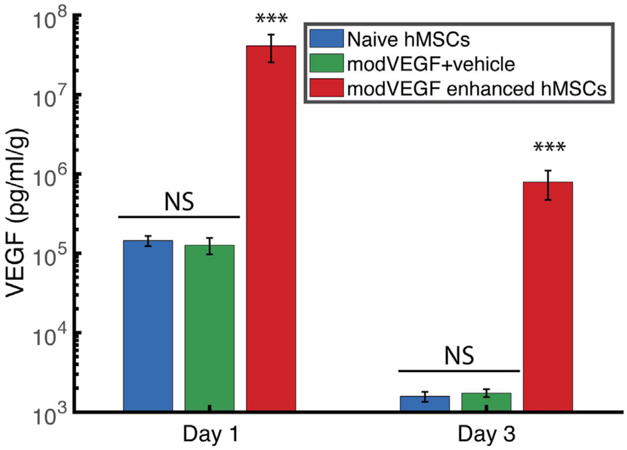

Figure 5. modRNA-enhanced hMSCs with modVEGF increase protein expression 200-fold compared with naive cells or naked modRNA alone at 1 day and approximately 500 times at 3 days after injections

NS, nonsignificant. Asterisks (***) indicate $p < 0.001$. Error bars represent the standard deviation.

1 mM EDTA at 1 μg/μL for use. Open reading frame sequences used for modRNA production were the same as previously described for EGFP and human VEGF-A165 (Table S2).[27]

hMSCs expansion

hMSCs were obtained from Lonza (Basel, Switzerland) together with the MSC-GM BulletKit (Lonza) and cultured according to the manufacturer's protocols in T175 flasks (Sarstedt, Nümbrecht, Germany). All experiments were performed on cells harvested before or on passage six.

modRNA transfection

Electroporation of hMSCs with mRNA encoding for GFP (modGFP) or VEGF (modVEGF) was conducted using the MaxCyte GT Transfection System. Briefly, cells were collected and washed in an electroporation buffer (HyClone). The cells were then mixed with modRNA to a total volume of 100 μL and electroporated in an OC-100 or OC-400 cuvettes. After initial testing, electroporation was conducted using an optimized program. The instrument settings are proprietary to MaxCyte, Inc. Cells were transferred to an incubator at 37°C and 5% CO_2 before resuspending them with the MSC-GM BulletKit and transferred them to culture flasks or immediately frozen in 10% DMSO.

Flow cytometry

GFP luminescence was characterized by flow cytometry on a Guava easyCyte HT flow cytometer (Luminex, Austin, TX, USA). The cells were harvested 24 h post electroporation and incubated in a flow cytometry buffer containing 10% fetal bovine serum, 1% BSA, and 0.5 mM EDTA. To assess viability, the hMSCs were stained with the Zombie Aqua Fixable Viability Kit (BioLegend, San Diego, CA, USA) according to the manufacturer's instructions. Results were analyzed using Guava InCyte Software (Luminex).

TW device pass-through

A volume of suspension containing 0.2 mL naive hMSCs were flushed through the TW device at three different cell concentrations (50,000, 30,000, and 20,000 cells/μL) and three different flow speeds (30, 120, and 240 s). Each speed and concentration was replicated three times. Enhanced cells with modVEGF and modGFP were flushed through the device at two different speeds (30 and 120 s). Non-electroporated hMSCs were used as controls.

ELISA in cell culture

For measuring the expression kinetics of modVEGF, the cell culture supernatant was collected at specified time points (24, 48, 72, 96, and 120 h after transfection), and concentrations of human VEGF-A protein were quantified using ELISA (R&D Systems, Inc., Minneapolis, MN, USA) according to the manufacturer's instructions. The optical density values of absorbance were measured on a microplate reader (ELX800; BioTek, USA).

Ethical considerations

All animal studies were conducted according to Karolinska Institutet guidelines for animal experiments and approved by the Regional Ethics Committee for Animal Research in Stockholm, Sweden (no. 1369-2021).

Swine experimental setup

Studies were conducted at the Karolinska Experimental Research and Imaging Center, Karolinska University Hospital, Stockholm. Fifteen female swine with weights between 32 and 42 kg were used in this study. Each animal fasted for 12 h with free access to water before the procedure. They were premedicated with intramuscular cepetor vet 1 mg/mL-zoletil 100 (Vetmedic/Virbac, Thirsk, UK) 0.8–1 mg/kg. Induction of anesthesia was conducted with propofol (Sandoz, Holzkirchen, Germany) 1–3 mg/kg and fentanyl (B. Braun, Melsungen, Germany) 2.5 μg/kg as an i.v. bolus dose. Maintenance of anesthesia was achieved with continuous infusion of propofol (10 mg/kg/min) and morphine (Meda, Solna, Sweden) (0.1–0.25 mg/kg/h) titrated to a moderate depth of anesthesia. No muscle relaxants were used during the experiment. We administered 75 mg amiodarone i.v. before the endovascular intervention and 5000 U heparin in positive pressure infusates.

Intubation using an endotracheal tube (7.0) was performed after induction of anesthesia and during spontaneous breathing. The animals were placed in a supine position and normo-ventilated by pressure-controlled ventilation with a Drager Apollo ventilator (Drägerwerk AK, Lübeck, Germany) with a total tidal volume of 10 mL/kg and with an inspiratory oxygen fraction (FiO_2) of 0.2143.

Figure 4. Endovascularly transplanted hMSCs to the myocardium using the TW device are found 24 h and 72 h post-injection in the swine heart

(A) Schematic image of experimental set up. (B) Number of cells on each slide at the injection sites at 24 h compared with 72 h after injections. (C) Immunofluorescent images stained with troponin, human nuclear antigen (HuN), and DAPI, showing hMSCs *in situ*. Dashed lines showing the TW device channel. (D) Macroscopic and microscopic images of the heart after injections. White arrows show injection sites.

Swine were euthanized 24 h or 72 h after the experiment by i.v. infusion of pentobarbital 100 mg/mL with 70% ethanol.

TW device

The specification, dimensions, and fabrication process of the TW device has been described previously.[36,45] The device features a superelastic nitinol tube with a sharp tip housed in a custom polytetrafluoroethylene catheter to shield the sharp tip during navigation. The mean outer diameter (OD) of the TW device is 246 μm and 413 μm with the polytetrafluoroethylene catheter. The tip is sharpened to minimize penetration force while resisting tip buckling. The depth-limiting collar, which prevents a deeper penetration than desired, was located 5 mm from the tip on all devices used in the experiments. Tip detachment was not used in the experiments.

Endovascular intervention

A Philips XD20 angiographic system (Philips Healthcare, Best, the Netherlands) was used for catheter placement and navigation. Vessel puncture of the femoral artery was performed using micropuncture technique (Merit Medical AB, Utah, USA) guided by ultrasonography (Siemens Acuson Sequoia 512 S Healthineers, Erlangen, Germany). Endovascular access was established with one 6-F introducer (Terumo, Tokyo, Japan) in the femoral artery. We used the arterial introducer for continuous monitoring of systemic arterial pressure. We placed a 5-F vertebral catheter (Merit Medical AB, Stockholm, Sweden) in the left ventricle of the heart and, within that, a 2.7-F Progreat catheter (Terumo, Tokyo, Japan) by fluoroscopic guidance. The TW device was advanced to the heart through these catheters.

Major complications to the intervention were defined as death from any cause, stroke, myocardial infarction, or tamponade.

Cell and modRNA injections

The swines received hMSCS, modRNA-enhanced hMSCs, or modVEGF in the left ventricle. Injections were aimed to be around the apex region of the heart. For the initial exploratory study, we injected 6 million naive hMSCs at six different locations. For the comparative study, we injected 3 million naive or modRNA-enhanced hMSCs at three different locations. We resuspended the cells in 200 μL of saline for all injections. The modVEGF was transfected in a sucrose-citrate buffer as this has previously been shown to be the most effective transfection method in rodents.[29,37] The sucrose-citrate buffer contained 200 μL sucrose in nuclease-free water (0.3 g/mL) and 200 μL citrate (0.1 M, pH 7; Sigma) mixed with 200 μL modRNA to a concentration of 0.3 μg/μL; 200 μL of the modRNA solution, i.e., 60 μg, was injected into the ventricle. Finally, we injected 100 μL of methylene blue to locate the injections postmortem.

Previous studies have shown peak VEGF mRNA expression occurs within 18–24 h post-injection.[27,37] Therefore, we selected this time point together with 72 h after injections to compare the various groups.

Tissue handling, immunohistochemistry, and protein quantification

When swine were euthanized, the heart was immediately harvested. The injection sites were located by macroscopic analysis and excised to approximately 0.5–1 cm^3 cubes. Next, we submerged the tissue into isopentane mixed with dry ice and subsequently stored the sample in a −80°C freezer.

hMSCs were visualized in the heart by immunofluorescence. Endo-to-epicardial 12-μm cryosections were taken using a Leica cryostat (CM 3000; Leica Instruments GmbH, Nussloch, Germany). Sections were stored at −20°C until staining. The frozen sections were fixed in 4% buffered paraformaldehyde for 5 min at room temperature, washed in PBS, and blocked with 10% normal goat serum, 1% bovine serum antigen, and 0.1% Triton X-100 in PBS for 1 h at room temperature. We incubated the sections overnight at +4°C with Human Nuclear antigen (HuN), which is found in human cells but not in swine, primary antibodies (Mab1281, Sigma-Aldrich, St. Louis, Missouri, United States) together with Troponin-T primary antibodies (clone 9C2.1, Sigma-Aldrich, St. Louis, MO, USA). Fluorescent goat anti-mouse secondary antibodies (Invitrogen, Waltham, MA, USA) were added for incubation at room temperature for 1 h, together with DAPI during the last 10 min. Images were acquired using a Zeiss confocal microscope (LSM700, Zeiss, Stuttgart, Germany), processed using the Zen software (Zeiss, Stuttgart, Germany), and analyzed using ImageJ (National Institutes of Health, Bethesda, MD, USA). We quantified the cells on each slide, using this count as a proxy to infer the overall cell population.

For analysis of adverse effects of the transplantation such as infarction, hematoxylin and eosin staining was performed according to Mayer's protocol.[46] Briefly, formalin-fixed slides were dipped in Mayer's hematoxylin and rinsed in tap water, followed by eosin staining. Next, slides were dehydrated through an ethanol dilution series. Finally, stained slides were evaluated using light microscopy and imaged using a Canon 7D (Canon, Ota City, Tokyo, Japan) mounted to the microscope.

ELISA was performed in order to quantify the VEGF-A levels in the heart. Cryopreserved heart tissue was pulverized and resuspended in Tissue Protein Extraction Reagent (T-PER, Thermo Fisher, Waltham, MA, USA) with Halt Protease Inhibitor Cocktail (Thermo Fisher). The solution was sonicated and then centrifuged at 13,000 rpm. The supernatant was collected and concentrated to 5 μg/μL using the QuickStart Bradford assay kit (BioRad, Hercules, CA, USA) according to the manufacturer's instructions. The concentrated solvent was collected and concentrations of human VEGF-A protein were quantified using the V-Plex Human VEGF kit (Mesoscale, Rockville, MD, USA) according to the manufacturer's instructions. The electrochemiluminescence was measured using a MESO quickplex instrument (Mesoscale).

Images and processing

Fluoroscopic images were extracted from raw DICOM files using OsiriX MD DICOM software (Pixmeo, Bernex, Switzerland) and

montages prepared in Adobe Photoshop 2022 (Adobe Systems, San Jose, CA, USA). Angiographies were windowed and subtracted using OsiriX MD and post-processed in Adobe Photoshop. Post processing included cropping and image enlargement. All modifications except cropping were performed on the entire image. Gross anatomy photographs were taken with Canon 7D camera (Canon, Ota City, Tokyo, Japan) and background was removed using Adobe Photoshop.

Statistical analysis

Descriptive statistics are presented as mean (± standard deviation) throughout except for the number of cells on slides, which is presented as median (±IQR). All data were assumed to be normally distributed because of the small sample size. Statistical comparisons were performed using ANOVA and Tukey's test for post hoc analysis. We fitted a repeated measures model and used repeated measures ANOVA for the comparison of multiple groups over several time points. To compute the coefficient of determination, data were fitted to a linear regression model, where we also obtained intercepts and F-statistics. Statistics were computed using MATLAB, version 2021b (MathWorks, Natick, MA, USA).

DATA AND CODE AVAILABILITY

The datasets generated and/or analyzed during the current study are available to download from github.com/Jonathan-Al-Saadi/petPig or from the corresponding author on reasonable request.

SUPPLEMENTAL INFORMATION

Supplemental information can be found online at https://doi.org/10.1016/j.omtm.2024.101225.

ACKNOWLEDGMENTS

We would like to thank Pellina Jansson, Sara Lysén, Luis Antonio Oyarzo Araya, and Ann-Christine Sandberg-Nordqvist for excellent logistic and technical assistance. This study was supported by the Söderberg Foundations and MedTechLabs.

AUTHOR CONTRIBUTIONS

J.A., N.W., K.C., J.L., and S.H. initiated the study. J.A., M.W., M.S., and R.G. carried out animal experiments. J.A., M.W., J.S., R.G., I.F., A.A., M.C., and N.W. conducted the *in vitro* experiments. K.C., J.L., N.W., and S.H. supervised the study. J.A. analyzed the data and wrote the first draft of the manuscript together with M.W. All authors have read and approved the manuscript.

DECLARATION OF INTERESTS

R.G., J.L., N.W., and S.H. are shareholders of SmartCella Holding AB that has the IP rights to the Extroducer (*trans*-vessel wall device).

REFERENCES

1. Tsao, C.W., Lyass, A., Enserro, D., Larson, M.G., Ho, J.E., Kizer, J.R., Gottdiener, J.S., Psaty, B.M., and Vasan, R.S. (2018). Temporal Trends in the Incidence of and Mortality Associated With Heart Failure With Preserved and Reduced Ejection Fraction. JACC. Heart Fail. *6*, 678–685. https://doi.org/10.1016/j.jchf.2018.03.006.
2. Groenewegen, A., Rutten, F.H., Mosterd, A., and Hoes, A.W. (2020). Epidemiology of heart failure. Eur. J. Heart Fail. *22*, 1342–1356. https://doi.org/10.1002/ejhf.1858.
3. Chien, K.R., Frisén, J., Fritsche-Danielson, R., Melton, D.A., Murry, C.E., and Weissman, I.L. (2019). Regenerating the field of cardiovascular cell therapy. Nat. Biotechnol. *37*, 232–237. https://doi.org/10.1038/s41587-019-0042-1.
4. Ishikawa, K., Weber, T., and Hajjar, R.J. (2018). Human Cardiac Gene Therapy. Circ. Res. *123*, 601–613. https://doi.org/10.1161/CIRCRESAHA.118.311587.
5. Kastrup, J., Jørgensen, E., Fuchs, S., Nikol, S., Bøtker, H.E., Gyöngyösi, M., Glogar, D., and Kornowski, R. (2011). A randomised, double-blind, placebo-controlled, multicentre study of the safety and efficacy of BIOBYPASS (AdGVVEGF121.10NH) gene therapy in patients with refractory advanced coronary artery disease: the NOVA trial. EuroIntervention. *6*, 813–818. https://doi.org/10.4244/EIJV6I7A140.
6. Menasché, P. (2018). Cell therapy trials for heart regeneration — lessons learned and future directions. Nat. Rev. Cardiol. *15*, 659–671. https://doi.org/10.1038/s41569-018-0013-0.
7. Garbern, J.C., and Lee, R.T. (2013). Cardiac stem cell therapy and the promise of heart regeneration. Cell Stem Cell *12*, 689–698. https://doi.org/10.1016/j.stem.2013.05.008.
8. Mandal, P.K., and Rossi, D.J. (2013). Reprogramming human fibroblasts to pluripotency using modified mRNA. Nat. Protoc. *8*, 568–582. https://doi.org/10.1038/nprot.2013.019.
9. Witman, N., Zhou, C., Grote Beverborg, N., Sahara, M., and Chien, K.R. (2020). Cardiac progenitors and paracrine mediators in cardiogenesis and heart regeneration. Semin. Cell Dev. Biol. *100*, 29–51. https://doi.org/10.1016/j.semcdb.2019.10.011.
10. Chong, J.J.H., Yang, X., Don, C.W., Minami, E., Liu, Y.-W., Weyers, J.J., Mahoney, W.M., Van Biber, B., Cook, S.M., Palpant, N.J., et al. (2014). Human embryonic-stem-cell-derived cardiomyocytes regenerate non-human primate hearts. Nature *510*, 273–277. https://doi.org/10.1038/nature13233.
11. Laflamme, M.A., Chen, K.Y., Naumova, A.V., Muskheli, V., Fugate, J.A., Dupras, S.K., Reinecke, H., Xu, C., Hassanipour, M., Police, S., et al. (2007). Cardiomyocytes derived from human embryonic stem cells in pro-survival factors enhance function of infarcted rat hearts. Nat. Biotechnol. *25*, 1015–1024. https://doi.org/10.1038/nbt1327.
12. Vagnozzi, R.J., Maillet, M., Sargent, M.A., Khalil, H., Johansen, A.K.Z., Schwanekamp, J.A., York, A.J., Huang, V., Nahrendorf, M., Sadayappan, S., and Molkentin, J.D. (2020). An acute immune response underlies the benefit of cardiac stem cell therapy. Nature *577*, 405–409. https://doi.org/10.1038/s41586-019-1802-2.
13. Chang, C., Yan, J., Yao, Z., Zhang, C., Li, X., and Mao, H.Q. (2021). Effects of Mesenchymal Stem Cell-Derived Paracrine Signals and Their Delivery Strategies. Adv. Healthcare Mater. *10*, 2001689. https://doi.org/10.1002/adhm.202001689.
14. Pittenger, M.F., Discher, D.E., Péault, B.M., Phinney, D.G., Hare, J.M., and Caplan, A.I. (2019). Mesenchymal stem cell perspective: cell biology to clinical progress. Npj Regen. Med. *4*, 22. https://doi.org/10.1038/s41536-019-0083-6.
15. Hare, J.M. (2019). A Phase I/II, Randomized, Double-Blinded, Placebo-Controlled Study of the Safety and Efficacy of Intramyocardial Injection of Autologous Human Mesenchymal Stem Cells (MSCs) in Patients with Chronic Ischemic Left Ventricular Dysfunction Secondary to Myocardial Infarction (MI) Undergoing Cardiac Surgery for Coronary Artery Bypass Grafting (CABG). (clinicaltrials.gov).
16. Mathur, A. (2021). The Effect of Intracoronary Reinfusion of Bone Marrow-Derived Mononuclear Cells(BM-MNC) on All Cause Mortality in Acute Myocardial Infarction. (clinicaltrials.gov).
17. Fischer, U.M., Harting, M.T., Jimenez, F., Monzon-Posadas, W.O., Xue, H., Savitz, S.I., Laine, G.A., and Cox, C.S. (2009). Pulmonary Passage is a Major Obstacle for Intravenous Stem Cell Delivery: The Pulmonary First-Pass Effect. Stem Cell. Dev. *18*, 683–692. https://doi.org/10.1089/scd.2008.0253.
18. Dunbar, C.E., High, K.A., Joung, J.K., Kohn, D.B., Ozawa, K., and Sadelain, M. (2018). Gene therapy comes of age. Science *359*, eaan4672. https://doi.org/10.1126/science.aan4672.
19. Fus-Kujawa, A., Prus, P., Bajdak-Rusinek, K., Teper, P., Gawron, K., Kowalczuk, A., and Sieron, A.L. (2021). An Overview of Methods and Tools for Transfection of Eukaryotic Cells in vitro. Front. Bioeng. Biotechnol. 9, 701031.
20. Ndeupen, S., Qin, Z., Jacobsen, S., Bouteau, A., Estanbouli, H., and Igyártó, B.Z. (2021). The mRNA-LNP platform's lipid nanoparticle component used in preclinical

vaccine studies is highly inflammatory. iScience *24*, 103479. https://doi.org/10.1016/j.isci.2021.103479.

21. Shirley, J.L., de Jong, Y.P., Terhorst, C., and Herzog, R.W. (2020). Immune Responses to Viral Gene Therapy Vectors. Mol. Ther. *28*, 709–722. https://doi.org/10.1016/j.ymthe.2020.01.001.
22. Chamberlain, K., Riyad, J.M., and Weber, T. (2017). Cardiac Gene Therapy with Adeno-Associated Virus-Based Vectors. Curr. Opin. Cardiol. *32*, 275–282. https://doi.org/10.1097/HCO.0000000000000386.
23. Lek, A., Wong, B., Keeler, A., Blackwood, M., Ma, K., Huang, S., Sylvia, K., Batista, A.R., Artinian, R., Kokoski, D., et al. (2023). Death after High-Dose rAAV9 Gene Therapy in a Patient with Duchenne's Muscular Dystrophy. N. Engl. J. Med. *389*, 1203–1210. https://doi.org/10.1056/NEJMoa2307798.
24. Chien, K.R., Zangi, L., and Lui, K.O. (2014). Synthetic Chemically Modified mRNA (modRNA): Toward a New Technology Platform for Cardiovascular Biology and Medicine. Cold Spring Harb. Perspect. Med. *5*, a014035. https://doi.org/10.1101/cshperspect.a014035.
25. Baden, L.R., El Sahly, H.M., Essink, B., Kotloff, K., Frey, S., Novak, R., Diemert, D., Spector, S.A., Rouphael, N., Creech, C.B., et al. (2021). Efficacy and Safety of the mRNA-1273 SARS-CoV-2 Vaccine. N. Engl. J. Med. *384*, 403–416. https://doi.org/10.1056/NEJMoa2035389.
26. Polack, F.P., Thomas, S.J., Kitchin, N., Absalon, J., Gurtman, A., Lockhart, S., Perez, J.L., Pérez Marc, G., Moreira, E.D., Zerbini, C., et al. (2020). Safety and Efficacy of the BNT162b2 mRNA Covid-19 Vaccine. N. Engl. J. Med. *383*, 2603–2615. https://doi.org/10.1056/NEJMoa2034577.
27. Zangi, L., Lui, K.O., von Gise, A., Ma, Q., Ebina, W., Ptaszek, L.M., Später, D., Xu, H., Tabebordbar, M., Gorbatov, R., et al. (2013). Modified mRNA directs the fate of heart progenitor cells and induces vascular regeneration after myocardial infarction. Nat. Biotechnol. *31*, 898–907. https://doi.org/10.1038/nbt.2682.
28. Drzeniek, N.M., Kahwaji, N., Schlickeiser, S., Reinke, P., Geißler, S., Volk, H.-D., and Gossen, M. (2023). Immuno-engineered mRNA combined with cell adhesive niche for synergistic modulation of the MSC secretome. Biomaterials *294*, 121971. https://doi.org/10.1016/j.biomaterials.2022.121971.
29. Sultana, N., Magadum, A., Hadas, Y., Kondrat, J., Singh, N., Youssef, E., Calderon, D., Chepurko, E., Dubois, N., Hajjar, R.J., and Zangi, L. (2017). Optimizing Cardiac Delivery of Modified mRNA. Mol. Ther. *25*, 1306–1315. https://doi.org/10.1016/j.ymthe.2017.03.016.
30. Ai, X., Yan, B., Witman, N., Gong, Y., Yang, L., Tan, Y., Chen, Y., Liu, M., Lu, T., Luo, R., et al. (2023). Transient secretion of VEGF protein from transplanted hiPSC-CMs enhances engraftment and improves rat heart function post MI. Mol. Ther. *31*, 211–229. https://doi.org/10.1016/j.ymthe.2022.08.012.
31. Yu, Z., Witman, N., Wang, W., Li, D., Yan, B., Deng, M., Wang, X., Wang, H., Zhou, G., Liu, W., et al. (2019). Cell-mediated delivery of VEGF modified mRNA enhances blood vessel regeneration and ameliorates murine critical limb ischemia. J. Contr. Release *310*, 103–114. https://doi.org/10.1016/j.jconrel.2019.08.014.
32. Sheng, C.C., Zhou, L., and Hao, J. (2013). Current Stem Cell Delivery Methods for Myocardial Repair. BioMed Res. Int. *2013*, 547902. https://doi.org/10.1155/2013/547902.
33. Thuijs, D.J.F.M., Kappetein, A.P., Serruys, P.W., Mohr, F.-W., Morice, M.-C., Mack, M.J., Holmes, D.R., Curzen, N., Davierwala, P., Noack, T., et al. (2019). Percutaneous coronary intervention versus coronary artery bypass grafting in patients with three-vessel or left main coronary artery disease: 10-year follow-up of the multicentre randomised controlled SYNTAX trial. Lancet Lond. Engl. *394*, 1325–1334. https://doi.org/10.1016/S0140-6736(19)31997-X.
34. Arnberg, F., Lundberg, J., Olsson, A., Samén, E., Jaff, N., Jussing, E., Dahlén, U., Nava, S., Axelsson, R., Ringdén, O., et al. (2016). Intra-arterial Administration of Placenta-Derived Decidual Stromal Cells to the Superior Mesenteric Artery in the Rabbit: Distribution of Cells, Feasibility, and Safety. Cell Transplant. *25*, 401–410. https://doi.org/10.3727/096368915X688191.
35. Lundberg, J., Södersten, E., Sundström, E., Le Blanc, K., Andersson, T., Hermanson, O., and Holmin, S. (2012). Targeted Intra-arterial Transplantation of Stem Cells to the Injured CNS is more Effective than Intravenous Administration: Engraftment is Dependent on Cell Type and Adhesion Molecule Expression. Cell Transplant. *21*, 333–343. https://doi.org/10.3727/096368911X576036.
36. Lundberg, J., Grankvist, R., and Holmin, S. (2019). The creation of an endovascular exit through the vessel wall using a minimally invasive working channel in order to reach all human organs. J. Intern. Med. *286*, 309–316. https://doi.org/10.1111/joim.12939.
37. Carlsson, L., Clarke, J.C., Yen, C., Gregoire, F., Albery, T., Billger, M., Egnell, A.-C., Gan, L.-M., Jennbacken, K., Johansson, E., et al. (2018). Biocompatible, Purified VEGF-A mRNA Improves Cardiac Function after Intracardiac Injection 1 Week Post-myocardial Infarction in Swine. Mol. Ther. Methods Clin. Dev. *9*, 330–346. https://doi.org/10.1016/j.omtm.2018.04.003.
38. AstraZeneca. (2021). A Randomized, Double-Blind, Placebo-Controlled, Multi-Centre, Sequential Design, Phase IIa Study to Evaluate Safety and Tolerability of Epicardial Injections of AZD8601 during Coronary Artery Bypass Grafting Surgery. clinicaltrials.gov.
39. Turnbull, I.C., Eltoukhy, A.A., Fish, K.M., Nonnenmacher, M., Ishikawa, K., Chen, J., Hajjar, R.J., Anderson, D.G., and Costa, K.D. (2016). Myocardial Delivery of Lipidoid Nanoparticle Carrying modRNA Induces Rapid and Transient Expression. Mol. Ther. *24*, 66–75. https://doi.org/10.1038/mt.2015.193.
40. Järveläinen, N., Halonen, P., Nurro, J., Hätinen, O.P., Korpela, H., Mäkinen, P., Gan, L.-M., Fritsche-Danielson, R., and Ylä-Herttuala, S. (2021). Citrate-Saline-Formulated mRNA Delivery into the Heart Muscle with an Electromechanical Mapping and Injection Catheter Does Not Lead to Therapeutic Effects in a Porcine Chronic Myocardial Ischemia Model. Hum. Gene Ther. *32*, 1295–1307. https://doi.org/10.1089/hum.2021.149.
41. Gonzalez-Fernandez, T., Sathy, B.N., Hobbs, C., Cunniffe, G.M., McCarthy, H.O., Dunne, N.J., Nicolosi, V., O'Brien, F.J., and Kelly, D.J. (2017). Mesenchymal stem cell fate following non-viral gene transfection strongly depends on the choice of delivery vector. Acta Biomater. *55*, 226–238. https://doi.org/10.1016/j.actbio.2017.03.044.
42. Guo, X., Wang, H., Li, Y., Leng, X., Huang, W., Ma, Y., Xu, T., and Qi, X. (2019). Transfection reagent Lipofectamine triggers type I interferon signaling activation in macrophages. Immunol. Cell Biol. *97*, 92–96. https://doi.org/10.1111/imcb.12194.
43. Ankrum, J.A., Ong, J.F., and Karp, J.M. (2014). Mesenchymal stem cells: immune evasive, not immune privileged. Nat. Biotechnol. *32*, 252–260. https://doi.org/10.1038/nbt.2816.
44. Zisa, D., Shabbir, A., Suzuki, G., and Lee, T. (2009). Vascular endothelial growth factor (VEGF) as a key therapeutic trophic factor in bone marrow mesenchymal stem cell-mediated cardiac repair. Biochem. Biophys. Res. Commun. *390*, 834–838. https://doi.org/10.1016/j.bbrc.2009.10.058.
45. Grankvist, R., Jensen-Urstad, M., Clarke, J., Lehtinen, M., Little, P., Lundberg, J., Arnberg, F., Jonsson, S., Chien, K.R., and Holmin, S. (2019). Superselective endovascular tissue access using trans-vessel wall technique: feasibility study for treatment applications in heart, pancreas and kidney in swine. J. Intern. Med. *285*, 398–406. https://doi.org/10.1111/joim.12841.
46. Bancroft, J.D., and Gamble, M. (2008). Theory and Practice of Histological Techniques (Elsevier health sciences).

OMTM, Volume *32*

Supplemental information

Endovascular transplantation of mRNA-enhanced mesenchymal stromal cells results in superior therapeutic protein expression in swine heart

Jonathan Al-Saadi, Mathias Waldén, Mikael Sandell, Jesper Sohlmér, Rikard Grankvist, Ida Friberger, Agneta Andersson, Mattias Carlsten, Kenneth Chien, Johan Lundberg, Nevin Witman, and Staffan Holmin

Supplemental figures

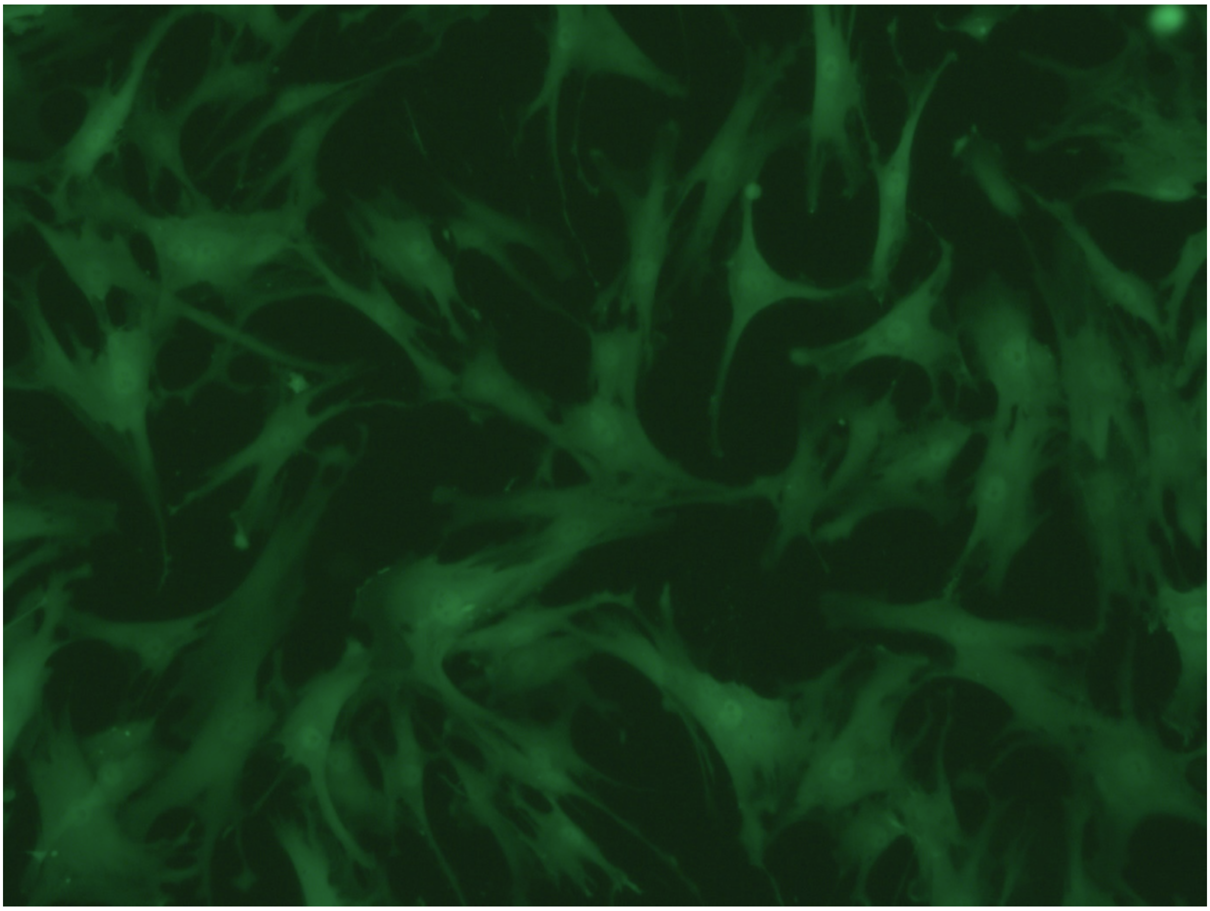

Figure S1. hMSCs electroporated with 20 μg modGFP per million cells at 7 days after electroporation.

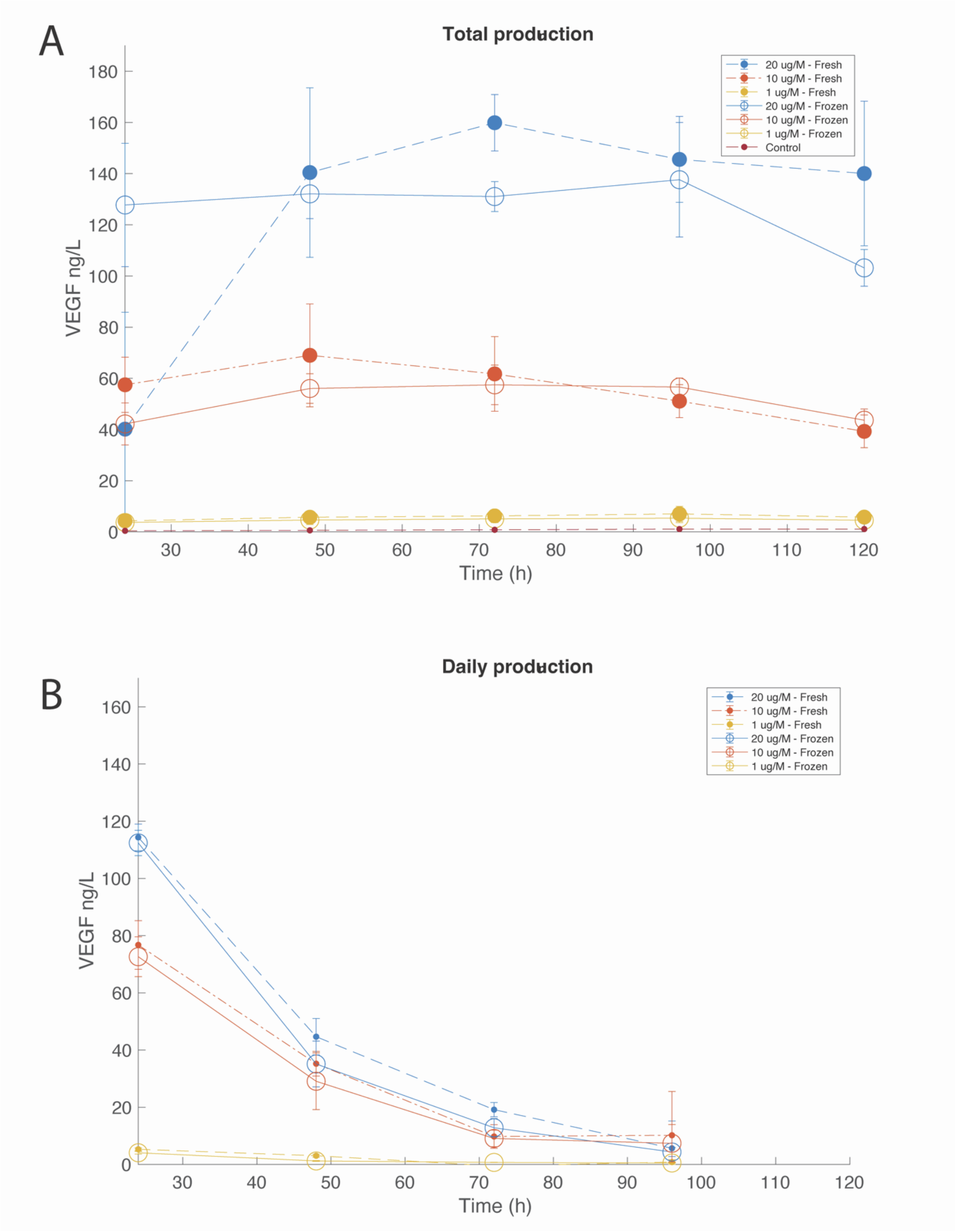

Figure S2. Levels of VEGF-A protein expression in cell culture supernatants of pre- and post-freeze-thawed hMSCs. A. Total production. B. Daily production. Triplicates in each group, $p > 0.05$.

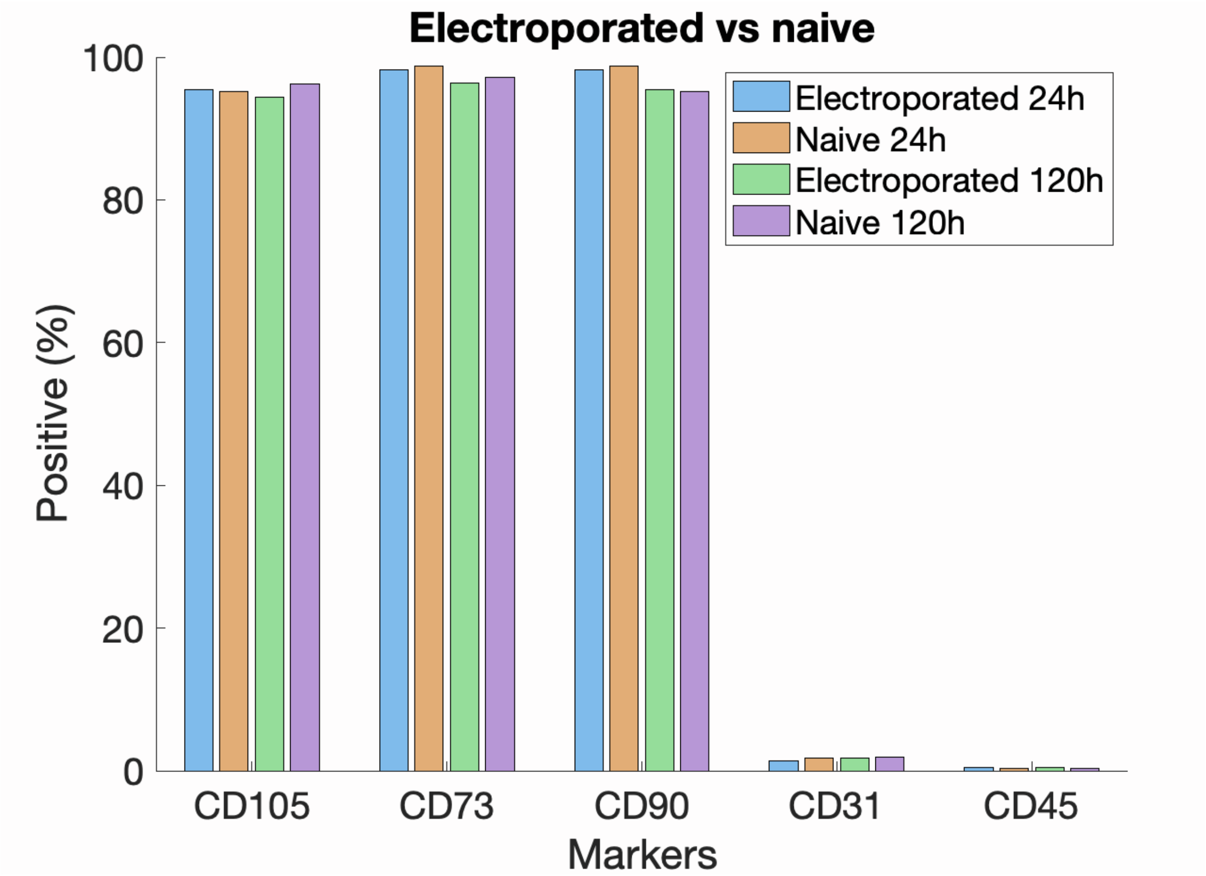

Figure S3. Quantification of hMSC markers pre- and post- electroporation monitored at 24h and 120h using flow cytometry. Triplicates in each group, $p > 0.05$.

Table S1. Flushed cells through the catheter at different speeds and concentrations.

Cell concentration (cells/uL)	Time after thaw (h)	Viability, mean (SD)	Flow speed (uL/s)	Proportion flushed, mean (SD)
30 000	6	88% (6%)	1.67	89% (8%)
50 000	0	86% (6%)	6.67	91% (7%)
	0	92 (6%)	1.67	80% (18%)
	0	94% (5%)	0.83	62% (15%)
20 000	0	89% (2%)	6.67	86% (7%)
	0	95% (5%)	1.67	61% (8%)
	0	95% (3%)	0.83	55% (9%)

Table S2. Open reading frame sequences used for modRNA production.

Name	Sequence
GFP	atggtgagcaagggcgaggagctgttcaccggggtggtgcccatcctggtcgagctggacgg cgacgtaaacggccacaagttcagcgtgtccggcgagggcgagggcgatgccacctacggca agctgaccctgaagttcatctgcaccaccggcaagctgcccgtgccctggcccaccctcgtg accaccctgacctacggcgtgcagtgcttcagccgctaccccgaccacatgaagcagcacga cttcttcaagtccgccatgcccgaaggctacgtccaggagcgcaccatcttcttcaaggacg acggcaactacaagacccgcgccgaggtgaagttcgagggcgacaccctggtgaaccgcatc gagctgaagggcatcgacttcaaggaggacggcaacatcctggggcacaagctggagtacaa ctacaacagccacaacgtctatatcatggccgacaagcagaagaacggcatcaaggtgaact

	tcaagatccgccacaacatcgaggacggcagcgtgcagctcgccgaccactaccagcagaac acccccatcggcgacggccccgtgctgctgcccgacaaccactacctgagcacccagtccgc cctgagcaaagaccccaacgagaagcgcgatcacatggtcctgctggagttcgtgaccgccg ccgggatcactctcggcatggacgagctgtacaagtaa
Human VEGF-A_{165}	atgaactttctgctgtcttgggtgcattggagccttgccttgctgctctacctccaccatgc caagtggtcccaggctgcacccatggcagaaggaggagggcagaatcatcacgaagtggtga agttcatggatgtctatcagcgcagctactgccatccaatcgagaccctggtggacatcttc caggagtaccctgatgagatcgagtacatcttcaagccatcctgtgtgcccctgatgcgatg cgggggctgctgcaatgacgagggcctggagtgtgtgcccactgaggagtccaacatcacca tgcagattatgcggatcaaacctcaccaaggccagcacataggagagatgagcttcctacag cacaacaaatgtgaatgcagaccaaagaaagatagagcaagacaagaaaatccctgtgggcc ttgctcagagcggagaaagcatttgtttgtacaagatccgcagacgtgtaaatgttcctgca aaaacacagactcgcgttgcaaggcgaggcagcttgagttaaacgaacgtacttgcagatgt gacaagccgaggcggtga
